# Analysis of the correlation between clinical nurses' professional quality of life and family care and organizational support

**DOI:** 10.3389/fpubh.2023.1108603

**Published:** 2023-02-22

**Authors:** Shan Xu, Dandan Ju, Ye Chen, Meiliyang Wu, Lan Wang, Xinxue Xi, Tieying Zeng

**Affiliations:** ^1^Department of Nursing, Tongji Medical College, Tongji Hospital, Huazhong University of Science and Technology, Wuhan, China; ^2^School of Nursing, Tongji Medical College, Huazhong University of Science and Technology, Wuhan, China

**Keywords:** quality of life, nurses, compassion satisfaction, burnout, nurse and patient

## Abstract

**Background and aim:**

Nurses' professional quality of life (ProQOL) is closely related to nursing life, and it is essential to clarify the professional quality of life of nurses and enhance it effectively. We aimed to explore the influence of family care and organizational support on the professional quality of life of clinical nurses and to improve the theoretical basis for improving the professional quality of life of clinical nurses in China.

**Methods:**

A single center, descriptive, cross-sectional design was used in this study. From February to April 2,022, 2,200 nurses from Tongji Hospital were selected as the study population, using the General Information Form, Family Care Scale, Professional Quality of Life Scale, Nurses' Sense of Organizational Support Questionnaire, and Work-Family Conflict Scale, and analyzing the relationship between professional quality of life and family care and organizational support among clinical nurses for correlation analysis as well as multiple linear regression to determine the factors affecting participants' Factors affecting ProQOL. *P* < 0.05 values were considered statistically significant.

**Results:**

The Cronbach coefficients of the scales were generally above 0.8, with good validity. All nurses had empathic satisfaction, burnout, and secondary trauma scores of 28.74 ± 3.83, 30.82 ± 3.45 and 29.40 ± 3.69, respectively, and correlation analysis, as well as multiple linear regression results, showed that the three dimensions of nurses' professional quality of life were associated with family care and organizational support (*P* < 0.05).

**Conclusions:**

The professional quality of life of nurses deserves to draw focused attention. The degree of family care and organizational support are predictive of professional quality of life, and nursing managers should pay attention to rationalizing tasks, pay attention to the physical and mental health of nursing staff, and improve the quality of life of nurses.

## Introduction

Professional quality of life (ProQOL) is the quality of life that those in the helping professions reap from their work, including compassion satisfaction and compassion fatigue (burnout and secondary trauma) (1, 2). ProQOL research in China started late and also involved a few departments. Previous studies also showed that the ProQOL of Chinese clinical front-line nurses was at an intermediate level, indicating that the professional quality of life situation of clinical nurses in China needs to be improved, especially the impact of family care and organizational support on nurses' professional quality of life needs to be understood (3, 4).

Researchers have different opinions about the factors affecting the professional quality of life of nurses; Research on the quality of professional life of nurses is increasing worldwide. In Europe, researchers such as Ruiz-Fernández have concluded that nursing professionals have increased levels of compassion fatigue and burnout but face certain factors that may affect the quality of professional life, among which is the relationship with the work environment (2). Vidal-Blanco et al. (5) believe that emotions are essential to improve the quality of work life of nurses and that interventions or training programs should be proposed to counteract emotional stress. In Asia, researchers such as Alshehry have linked nurses' experiences of workplace civility to ProQOL (6); Cruz has highlighted the importance of optimism and positive coping in ensuring a high quality of life for nurses (7). In China, Wang et al. (8) believe that the quality of professional life of Chinese nurses is generally poor, and that poor sleep quality, low job satisfaction, and long working hours are associated with burnout, and also suggest the implementation of targeted strategies to reduce burnout and secondary stress in nurses; Yu et al. (9) conducted a more in-depth investigation of oncology nurses and found higher fatigue and burnout in oncology nurses with years of nursing experience, secondary hospital work, and passive coping styles due to the specificity of oncology nurses. The results may provide clues to help managers identify nurses with low levels of ProQOL. All of these factors mentioned above can affect nurses' professional quality of life (2, 10). Meanwhile Khatatbeh et al. (11) hypothesized a model by integrating two theoretical models: the compassion satisfaction-compassion fatigue and the empowerment model, and the integrated model could also explain most of the factors of professional quality of life and burnout.

Due to the specific nature of the work of nursing staff, their characteristics and surroundings impact the quality of their professional life. Guerra et al. (12) found in an RCT that healthcare for caregivers can promote health, improve quality of life and reduce stress and that appropriate scientific interventions for caregivers are, therefore, necessary; Adolfo's survey of 427 Saudi nurses found that quality of professional life was correlated with gender, marital status and that male nurses had a better quality of life (13). In contrast, male nurses were more tolerant of workplace bullying and caused less psychological damage (14).

For the professional quality of life of nurses, the main categories are compassionate satisfaction, burnout and secondary trauma. Compassionate satisfaction (CS) is considered to be the satisfaction that healthcare professionals experience when they perform their jobs properly, which also includes satisfaction with their relationships with colleagues and a sense that the work they perform is socially valuable (15); Burnout (BO) is defined as a syndrome of emotional exhaustion, depersonalization, and lack of personal fulfillment at work, characteristics that develop as a result of continuous exposure to work stressors (16); secondary trauma (ST) is thought to result from caring for a patient who has experienced a traumatic or stressful event, leading to emotional symptoms or distress in the carer (17).

Previous studies rarely mentioned the impact of family care or organizational support on nurses' professional quality of life, which plays an essential role in nurses' psychology, attitude, and behavior (13, 18). This emotional support is an intangible support that satisfies clinical nurses' psychological sense of family and unit belonging. Family care and unit support also positively affect further career improvement. We aimed to explore the impact of family care or organizational support on nurses' professional quality of life and to intervene in advance.

## Materials and methods

### Design

A single center, descriptive, cross-sectional design was used.

### Research nurse subjects

A questionnaire was administered to nursing staff in 18 departments, and before completing the questionnaire, the researcher briefly introduced the type of questionnaire. The estimated time for completion was 15 min. Participation was voluntary and anonymous, and participants were informed of the purpose of the study. A total of 2,383 questionnaires were received, of which 2,200 were valid. A total of 2,200 nurses from Tongji Hospital, Tongji Medical College, Huazhong University of Science and Technology were selected as the study population from February to April 2022 using convenience sampling. We used strict inclusion and exclusion criteria, and the inclusion criteria were (1) obtaining a Chinese nursing license and completing registration, (2) practicing clinical nursing for at least 1 year, (3) having no impairment in communication skills and no psychiatric and psychiatric disorders (4) voluntarily participated in this study. Exclusion criteria were (1) unofficially enrolled nurses (*n* = 108), (2) nurses not on duty during the survey period (*n* = 56) (3) previous psychiatric disorders (*n* = 22). After exclusion, the remaining sample size was 2014. This study complied with the requirements of the Declaration of Helsinki.

### Survey tools and forms

The general information questionnaire was designed in consultation with several investigators. It included age, ethnicity, gender, department, technical title, type of position, years of experience, number of night shifts, highest education, marital status, daily commuting time, hobbies, history of chronic diseases, hypertension, diabetes, history of stroke, and sleep condition.

Snulkstein, MD, designed the Family Care Scale in 1978 as a self-assessment APGAR questionnaire (19) that included five dimensions: Adaptation, Partnership, Growth, Affection, and Resolve (Supplementary Table 1).

Stamm developed the Professional Quality of Life Scale. The scale consists of three dimensions: compassionate satisfaction, job burnout, and secondary trauma, and each dimension contains 10 items, which are assigned a score of 1–5 from “never” to “always.” The sum of the scores of each dimension is low level, 23–41 is medium level, and ≥42 is high level (Supplementary Table 2).

The nurse perception of organizational support questionnaire was developed by Haozen Wang. It has 1 dimension and 15 items, with a Cronbach coefficient of 0.985, a half reliability of 0.909, and a retest reliability of 0.812. The Likert 5-point scale was used, with scores from 1 to 5 representing “strongly disagree” to “strongly agree,” respectively. “The higher the score, the higher the perceived level of organizational support of the nurses (see Supplementary Table 3 for the questionnaire). The Work-Family Conflict Scale (WFCS), developed by Hauk and Chodkiewicz (20), has 2 dimensions (work-court conflict and family-work conflict) and 10 items on a 5-point Likert scale (Supplementary Table 4).

All the above questionnaires were translated into Chinese in advance (21), and the consent and cooperation of the hospital and each department were obtained. A uniformly trained surveyor conducted the survey. The questionnaires were filled out at a uniform time and collected by the surveyor on the spot and checked for content, and if there were any missing items, they were completed on the spot.

In this study, 2,200 questionnaires were distributed, and 2,014 valid questionnaires were returned, with a valid return rate of 91.5%.

### Data analysis

We used spss version 25.0 (SPSS Inc., Chicago, IL, USA) to analyze the data. Data regarding demographic characteristics, professional level, and other scales such as ProQOL were analyzed using descriptive statistics, including frequencies, percentages, means, standard deviations, quartiles, and standardized scores. We conducted t-tests, rank sum tests, and one-way ANOVAs for all scale variables based on participants' demographic and work-related characteristics. Correlation analyses and multiple linear regression were performed to determine the factors affecting participants' ProQOL. *P* < 0.05 values were considered statistically significant.

### Ethical statement

This study was approved by the Ethics Committee of the Wuhan Tongji Hospital. Approval number TJ-JRB20220923 [2022/08/16], all data managed by our team of data statisticians.

## Results

### General variables for clinical nurses

Complete data were collected from a total of 2,014 nurses in this study. The overall age was 33.4 ± 8.3 years, of which 69 (3.4%) were male, and 1,945 (96.6%) were female; 1,055 (52.4%) had the technical title of nurse practitioner; the majority of nursing staff were in clinical positions (1,644), accounting for 81.4%; the number of years of work was 11.2 ± 7.2 years; the average number of night shifts per year was 60.2; the Most of the nursing staff were undergraduate 1,897, accounting for 94.2%; nursing staff with a history of hypertension, diabetes, and stroke were the minority; 872 nursing staff had sleep problems, accounting for 43.3%, details of which are shown in Table 1.

**Table 1:** Basic information for clinical nurses (*n* = 2,014).

		**Whole cohort (*n* = 2,014)**
Age†	Year	33.4 ± 8.3
**Ethnicity**		
	Han Chinese	1,956 (97.1)
	Ethnic Minorities	58 (2.9)
**Gender**		
	Female	1,945 (96.6)
	Male	69 (3.4)
**Technical titles**		
	Nurse	155 (7.7)
	Nurse Practitioner	1,055 (52.4)
	Nurse-in-charge	749 (37.2)
	Associate Professor or Professor of Nursing	55 (2.7)
**Position type**		
	Non-clinical positions	370 (18.4)
	Clinical positions	1,644 (81.6)
Working years†		11.2 ± 7.2
Number of night shifts per year†		60.5 ± 34.1
**Highest degree**		
	Master or above	111 (5.5)
	Undergraduate	1,897 (94.2)
	College or below	6 (0.3)
**Marital status**		
	Married	1,483 (73.6)
	Unmarried	531 (26.4)
**Daily commuting time**		
	< 0.5 h	317 (15.7)
	0.5–1.0 h	705 (35.0)
	1.0–1.5 h	473 (23.5)
	>1.5 h	519 (25.8)
**Interests**		
	No	862 (42.8)
	Yes	1,152 (57.2)
**Hypertension**		
	No	1,958 (97.2)
	Yes	56 (2.8)
**Chronic medical history**		
	No	1,624 (80.6)
	Yes	390 (19.4)
**Diabetes**		
	No	1,998 (99.2)
	Yes	16 (0.8)
**Stroke**		
	No	2,013 (100.0)
	Yes	1 (0.0)
**Sleeping conditions**		
	Normal	1,142 (56.7)
	Abnormal	872 (43.3)

The values in parentheses are percentages unless indicated otherwise.

^†^Mean (standard deviation).

### Reliability test of the scale and the scores of each scale

In this study, the internal consistency of Cronbach's alpha coefficient was used to evaluate the reliability of the questionnaire. According to the results of the scales, the Cronbach's alpha coefficient for the Nurses' Professional Quality of Life General Scale was 0.931, the coefficient for the Family Support Scale was 0.905, the coefficient for the Organizational Support Scale was 0.985, and the Family Work Conflict Scale was 0.903, as detailed in Supplementary Table 5. The Nurses' Professional Quality of Life scores for empathic satisfaction was 28.74 ± 3.83, 30.82 ± 3.45 for fatigue, and 29.40 ± 3.70 for secondary trauma; the rest are detailed in Table 2.

**Table 2:** Clinical nurses' scores on each scale and on each dimension (*n* = 2,014).

**Various items**	**Dimensionality**	**Score**
Nurse professional quality of life scale	—	−
	Compassion satisfaction	28.74, 3.83
	Burnout	30.82, 3.45
	Secondary trauma	29.40, 3.70
Family care scale	—	7.27, 2.61
Organizational support scale	—	58.84, 11.29
Work-family conflict scale	—	27.17, 7.21

### Single-factor analysis of nurses' professional quality of life

The results using *t*-test or one-way ANOVA showed that for Compassion satisfaction, there was a statistical difference in scores for different marital statuses by highest education (*P* < 0.05); for burnout, there was a statistical difference in scores for different marital statuses and different sleep status (*P* < 0.05); for Secondary trauma, there were statistically significant differences in scores by highest education and by marital status (*P* < 0.05). For the rest of the scores, please see Table 3.

**Table 3:** Nurses' professional quality of life for univariate analysis (*n* = 2,014).

**Various items**	**Compassion satisfaction**	**Burnout**	**Secondary trauma**
**Gender**			
Male	28.50 ± 4.70	31.50 ± 3.55	29.97 ± 4.01
Female	29.00 ± 3.80	30.80 ± 3.44	29.38 ± 3.68
*t*	1.266	1.534	1.185
*P*	0.220	0.125	0.236
**Technical titles**			
Nurse	28.00 ± 3.83	30.49 ± 3.49	28.77 ± 3.83
Nurse Practitioner	28.71 ± 3.79	30.62 ± 3.41	29.24 ± 3.66
Nurse-in-charge	29.00 ± 3.90	31.01 ± 3.44	29.58 ± 3.67
Associate Professor or Professor of Nursing	29.89 ± 3.14	32.75 ± 3.40	31.87 ± 3.25
F	1.503	2.352	1.405
P	0.220	0.125	0.236
**Position type**			
Non-clinical positions	28.34 ± 3.38	31.17 ± 3.19	29.67 ± 3.56
Clinical positions	28.71 ± 3.88	30.68 ± 3.40	29.26 ± 3.66
*t*	3.334	4.444	5.406
*P*	0.122	0.321	0.287
**Highest degree**			
Master or above	29.49 ± 3.58	28.50 ± 2.88	28.17 ± 6.55
Undergraduate	28.70 ± 3.84	30.79 ± 3.43	29.35 ± 3.66
College or below	26.67 ± 3.39	31.38 ± 3.66	30.25 ± 3.96
*F*	3.100	2.887	3.452
*P*	0.045	0.056	0.032
**Marital status**			
Married	28.88 ± 3.84	30.99 ± 3.45	29.55 ± 3.68
Unmarried	28.30 ± 3.73	30.30 ± 3.41	28.92 ± 3.72
*t*	−2.914	−3.824	−3.268
*P*	0.004	0.001	0.001
**Chronic medical history**			
No	28.75 ± 3.88	30.77 ± 3.49	29.42 ± 3.67
Yes	28.68 ± 3.58	31.00 ± 3.27	29.33 ± 3.77
*t*	0.335	−1.191	0.392
*P*	0.737	0.234	0.695
**Sleeping conditions**			
No	28.74 ± 3.58	30.68 ± 3.27	29.30 ± 3.69
Yes	28.73 ± 4.13	31.00 ± 3.27	29.53 ± 3.70
*t*	0.075	−2.044	−1.400
*P*	0.940	0.041	0.162

Two groups of data conform to normal distribution using *t*-test, multiple groups of data conform to normal distribution using ANOVA.

### Scores on various scales between different sections

We show the scores of different sections on compassion satisfaction, burnout, secondary trauma, family support, life satisfaction, organizational support, and work-family conflict. The results showed that for family care, ICU and infection units scored the lowest; for empathy satisfaction, outpatient clinics, medical and technical departments, and operating rooms scored the lowest; for burnout, medical and technical departments, outpatient clinics, and surgery ranked the top three; for secondary trauma, medical and technical departments and surgery ranked the top two; and for work-family conflict, infection units, internal medicine, and emergency medicine ranked the top three. For the rest, see Supplementary Table 6.

### Correlation between family care and organizational support and three dimensions of professional quality of life

Pearson's correlation analysis showed that burnout, secondary trauma, family care, life satisfaction, organizational support, and work-family conflict were positively correlated with empathic satisfaction; family care was negatively correlated with work-family conflict (*r* = −0.406, *P* < 0.01) and positively correlated with several other factors; organizational support was negatively correlated with work-family conflict (*r* = −0.424, *P* < 0.01). There was no significant correlation between work-family conflict and secondary trauma. The remaining details are shown in Table 4.

**Table 4:** Correlation analysis of nurses' family care, organizational support and nurses' professional quality of life.

	**SC**	**Burnout**	**ST**	**FC**	**LS**	**OS**	**WFC**	**SOC**
SC	1	0.576^**^	0.520^**^	0.060^**^	0.171^**^	0.135^**^	0.046^*^	0.145^**^
Burnout	0.576^**^	1	0.629^**^	0.179^**^	0.320^**^	0.332^**^	−0.141^**^	0.219^**^
ST	0.520^**^	0.629^**^	1	0.141^**^	0.286^**^	0.205^**^	−0.028	0.179^**^
FC	0.60^**^	0.179^**^	0.141^**^	1	0.474^**^	0.362^**^	−0.356^**^	0.273^**^
LS	0.171^**^	0.320^**^	0.286^**^	0.474^**^	1	0.529^**^	−0.406^**^	0.252^**^
OS	0.135^**^	0.332^**^	0.205^**^	0.362^**^	0.529^**^	1	−0.424^**^	0.193^**^
WFC	0.046*	−0.141^**^	−0.028	−0.356^**^	−0.406^**^	−0.424^**^	1	−0.177^**^
SOC	0.145^**^	0.219^**^	0.179^**^	0.273^**^	0.252^**^	0.193^**^	−0.177^**^	1

^*^means *P* < 0.05; ^**^means *P* < 0.01.

SC, Compassion satisfaction; ST, Secondary trauma; FC, Family Care; LS, Life satisfaction; OS, Organizational Support; WFC, Work-family conflict; SOC, Sense of mental consistency.

### Establishing a multiple stepwise regression analysis of family caring degree, organizational support and other factors on nurses' professional quality of life

To further investigate the effects of family caring and organizational support on the professional quality of life of hospital nurses, multiple stepwise regression analyses were conducted using the variables collected from the general data (gender, age, highest education, title, etc.), scores on the family caring, organizational support, and work-family conflict scales as independent variables and scores on the dimensions of professional quality of life as dependent variables. The results showed that family care, organizational support, and work-family conflict were associated with all three dimensions of professional quality of life and were statistically different (*P* < 0.05), as detailed in Tables 5–7.

**Table 5:** Multiple stepwise regression results of nurses' family caring, perception of organizational support, and work-family conflict on compassion satisfaction (*n* = 2,014).

**Various items**	**B-value**	**β-value**	***t*-value**	***P*-value**	**Tolerance**	**VIF**
Constant	24.628		28.608	0.001		
Position type	−0.423	−0.094	−4.283	0.001	0.994	1.006
Family Care Scale	0.060	0.041	1.705	0.048	0.818	1.222
Organizational Support Scale	0.058	0.171	6.856	0.001	0.767	1.304
Work-Family Conflict Scale	0.071	0.133	5.359	0.001	0.772	1.295

*R*^2^ = 0.420, adjusted- *R*^2^ = 0.671, *F* = 21.856, *P* < 0.001.

**Table 6:** Multiple stepwise regression results of nurses' family caring, perception of organizational support, and work-family conflict on burnout (*n* = 2,014).

**Various items**	**B-value**	**β-value**	***t*-value**	***P*-value**	**Tolerance**	**VIF**
Constant	23.554		36.001	0.001		
Working years	0.049	0.103	4.921	0.001	0.995	1.005
Family Care Scale	0.093	0.071	3.062	0.002	0.819	1.221
Organizational Support Scale	0.097	0.317	13.336	0.001	0.768	1.301
Work-Family Conflict Scale	0.012	0.025	1.045	0.029	0.770	1.299

*R*^2^ = 0.525, adjusted- *R*^2^ = 0.523, *F* = 71.731, *P* < 0.001.

**Table 7:** Multiple stepwise regression results of nurses' family caring, perception of organizational support, and work-family conflict on secondary trauma (*n* = 2,014).

**Various items**	**B-value**	**β-value**	***t*-value**	***P*-value**	**Tolerance**	**VIF**
Constant	23.006		32.394	0.001		
Family care scale	0.140	0.099	4.179	0.001	0.819	1.221
Organizational support scale	0.069	0.210	8.474	0.001	0.769	1.301
Work-family conflict scale	0.049	0.096	3.899	0.001	0.773	1.294

*R*^2^ = 0.530, adjusted- *R*^2^ = 0.540, *F* = 38.420, *P* < 0.001.

## Discussion

In recent years, domestic research on nurses' professional quality of life has been gradually carried out. However, fewer departments are currently involved, and the sample size needs to be increased. Scholars in Europe and the United States have researched Pro-QOL earlier and involved multiple regions and specialties (22–26). Compared with Europe and the United States, clinical nurses' professional quality of life in China is relatively low. The situation needs to be improved, and there is more room for improvement. There are three main dimensions of professional quality of life, and this study aimed to assess the relationship between nurses' family care and organizational support and the three dimensions. Also, our study included more than 2,000 complete nurses, the largest sample size of a single-center study.

Our results show that Cronbach's coefficients of all scales were above 0.8, with good reliability. Also, in terms of scores, the three dimensions were generally at a moderate level, which is more consistent with most studies in Asia. A multicenter study by Ma suggested (27) that the professional quality of life of nurses in second- and third-level hospitals in Heilongjiang Province, China, was at a moderate level and should be improved by nursing managers, medical institutions, and other factors influencing the professional quality of life of nurses; Matsuishi et al. (28) conducted a Japanese nurses' professional quality of life, and the mean scores of the three dimensions were also around 30, which is basically consistent with our study and similar to previous reports assessing all nurses in Japan. Nurses in Europe and the United States generally scored higher, and Kelly (25) conducted a professional quality of life questionnaire survey of 491 nurses in a teaching hospital in the United States with an average Compassion Satisfaction score of 40.51 and a literature review by Flarity et al. (29) suggested that training can be effective in preventing and treating Compassion Satisfaction. The Asian region should adopt more interventions and focus on the factors affecting the professional quality of life.

According to the correlation results, the three dimensions of nurses' professional quality of life were associated with family care, life satisfaction, organizational support, and work-life and were statistically significant; also, the multivariate linear results showed that all three dimensions were associated with FC and OS. The family care index included adaptability, cooperation, maturity, emotionality, and intimacy. Adaptability is the ability to use internal and external family resources to solve problems when the family is in crisis; cooperation is the degree to which family members share responsibilities and make decisions together; maturity is the degree of physical and mental maturity and self-actualization achieved by family members through mutual support, and affectivity is the degree to which family members love each other. For nurses with a high degree of family caring, it indicates good family functioning, timely and effective use of resources, and a positive approach to coping with work stress. Piotrkowska et al. (30, 31) concluded that nurses' life satisfaction was significantly correlated with family life and that improving family care could lead to greater satisfaction in the life domain, improving nurses' professional quality of life.

Organizational support and professional quality of life correlation reference explanation: the sense of organizational support refers to the nursing staff's own perceived organizational support for work, which belongs to the work environment factors; perceived organizational support can meet nurses' emotional needs and keep them enthusiastic about departmental nursing, which helps to enhance nurses' professional wellbeing. Bobbio et al. (32) researchers concluded that strong nursing leadership could enhance trust in the organization, that trust can retain nursing staff, and that a good sense of organizational support can reduce nurses' work stress. Eisenberger et al. (33) also concluded that excellent organizational support and supervisory support could lead to an increased sense of belonging and, ultimately, job retention; Liu et al. (34) found that organizational support mediated the relationship between workplace violence, job satisfaction, burnout and propensity to leave in a survey of nurses' intention to leave in Chinese tertiary hospitals, and had a significant adverse effect on the propensity to leave. Therefore, nursing managers should understand the importance of organizational support and establish reasonable incentives to reduce the propensity to leave. Therefore, we need to implement a reasonable compensation and performance incentive system and try our best to provide support in all aspects, such as human, material, and financial resources, so that nurses can really experience the support of the hospital and thus enhance the sense of organizational support.

There are several limitations to this study; firstly, our data came from a large tertiary hospital in China, and the nurses who participated in the survey were likely to be more educated, more satisfied with their lives and jobs, and more financially affluent. It is important to compare the nurses in our center with those in other centers. Secondly, there was some selection bias as other centers, or non-tertiary hospital data did not validate it. In the future, we should expand the sample size, especially for samples from different centers. Thirdly, we used a convenience sample, which causes some bias.

## Conclusion

Therefore, nurses' professional quality of life is closely linked to family care and organizational support. Clinical nurses actively seek and take advantage of family care. Nursing managers focus on the timely identification of controllable factors for improvement, adopt appropriate intervention strategies, and give organizational support. Improve the nursing work environment and provide them with abundant work resources; give nurses more opportunities for further training and education. At the same time, the training and assessment of various nursing operation skills should be strengthened to improve nurses' professional and technical skills and independent working ability and provide nurses with a comprehensive development platform so they can experience the value of their work.

## Data availability statement

The original contributions presented in the study are included in the article/Supplementary material, further inquiries can be directed to the corresponding author.

## Ethics statement

This study was approved by the Ethics Committee of the Wuhan Tongji Hospital. Approval number TJ-JRB20220923 [2022/08/16]. Written informed consent from the participants was not required to participate in this study in accordance with the national legislation and the institutional requirements.

## Author contributions

SX: conceptualization, methodology, software, and writing–original draft preparation. DJ: conceptualization and methodology. YC: methodology and software. MW: data curation and writing–reviewing and editing. LW: visualization and investigation. XX: supervision. TZ: writing–reviewing and editing. All authors contributed to the article and approved the submitted version.
