# Supplementary material for: Analysis of the correlation between clinical nurses' professional quality of life and family care and organizational support
Source: Front Public Health. 2023 Feb 22;11:1108603. doi: 10.3389/fpubh.2023.1108603 (PMC9992405; doi:10.3389/fpubh.2023.1108603)
Supplement: Supplementary file 1 [file Table_1.pdf]

Supplementary Table 1. Family Care Scale

		Often	Sometimes	Rarely
1	When I have a problem, I can get satisfactory help from my family			
2	I'm happy with the way my family talks to me about things and shares problems			
3	My family is receptive and supportive when I want to pursue new activities or developments			
4	I am satisfied with the way my family shows concern and love for my emotions (joy, anger, sadness and happiness)			
5	I'm happy with the way my family spends time with me			

		经常这样	有时这样	几乎很少
1	当我遇到问题时，可以从家人得到满意的帮助			
2	我很满意家人与我谈论各种事情以及分担问题的方式			
3	当我希望从事新的活动或发展时，家人都能接受且基于支持			
4	我很满意家人对我的情绪（喜、怒、哀、乐）表示关心和爱护的方式<br>补充说明：_______________			
5	我很满意家人与我共度时光的方式<br>补充说明：_______________			
