# Supplementary material for: Analysis of the correlation between clinical nurses' professional quality of life and family care and organizational support
Source: Front Public Health. 2023 Feb 22;11:1108603. doi: 10.3389/fpubh.2023.1108603 (PMC9992405; doi:10.3389/fpubh.2023.1108603)
Supplement: Supplementary file 2 [file Table_2.pdf]

Supplementary Table 2. Professional Quality of Life Scale

	Never	Rarely	Sometimes	Often	Always
1.I am happy					
2. My mind is often filled with a person I have cared for before					
3. Nursing gives me a sense of satisfaction					
4. I feel connected to others (patients, colleagues, friends, etc.)					
5. Sudden sounds can startle me					
6. Being with people who need my care makes me feel refreshed					
7. I find it difficult to separate my personal life from my nursing work					
8. I can't work productively because I have lost sleep due to a traumatic experience of caring for someone					
9. I think I've been affected by the severely traumatized patients I care for					
10. Working as a nurse made me feel stuck in a rut					
11. Because my nursing behavior makes me nervous about a lot of things					
12. I like to work as a nurse					
13. I feel frustrated because of the traumatic experiences of the patients I care for					
14. I felt like I was experiencing the same trauma that the severely traumatized patients I was caring for had experienced					
15. I have faith to support me					

16. I feel good about my ability to keep up with advances in nursing technology and nursing policy					
17. I am the person I want to be					
18. I am satisfied with my work					
19. As a nurse, I feel exhausted					
20. I have happy thoughts about the people I care for and how I care for them					
21. As my own workload seemed endless, I felt unable to cope					
22. I believe that through my hard work, I will make a difference					
23. I avoid certain activities or situations because they remind me of my horrible experiences caring for patients					
24. I am proud to be able to care for others					
25. Because of the trauma patients I care for, I have intrusive and frightening thoughts					
26. I feel "deep" in the swamp of the system					
27. I think I am a successful nurse					
28. I don't remember the important parts of my work that relate to trauma patients					
29. I am a very caring person					
30. I am happy to have chosen to work as a nurse					

Supplementary Table 2. Professional Quality of Life Scale

	从未有过	很少	有些时候	经常如此	总是如此
1、我是快乐的					
2、我脑中常充满一个以前我所护理过的人					
3、护理让我得到满足感					
4、我感到与他人（患者、同事、朋友等）有关联					
5、突如其来的声音会让我感到惊吓					
6、与需要我护理的人在一起，让我感到神采奕奕					
7、我发现将我个人生活与护士工作分开是困难的					
8、我因护理过某个人的创伤性经历而失眠，所以工作不能富有成效					
9、我想我已经被那些我所护理的严重创伤患者所影响					
10、从事护士的工作让我感到陷入了困境					
11、因为我的护理行为，让我对很多事情感到紧张					
12、我喜欢从事护士的工作					
13、因为我护理的患者的创伤性经历，我感到沮丧					
14、我觉得我仿佛经历了那些曾经护理的严重创伤患者有过的创伤					
15、我有信念支持着我					
16、我对自己能够跟上护理技术和护理政策的进步而感到高兴					
17、我就是我想要成为的人					
18、我对自己的工作感到满意					
19、作为一名护士，我感到筋疲力尽					
20、对于那些我护理的人和我如何护理他们，我有开心的想法					
21、由于自己的工作负荷似乎无止境，我感到无法应对					
22、我相信通过我的努力工作，我会有所作为					
23、我避免某些活动或情况，因为他们让我想起我护理患者的可怕经历					
24、我为自己能够护理别人而感到自豪					

25、因为我护理的创伤患者，我有被侵扰和令人恐惧的想法					
26、我觉得"深陷"于制度的沼泽					
27、我认为我是一个成功的护士					
28、我不记得我工作中那些和创伤患者有关的重要部分					
29、我是一个非常有爱心的人					
30、能够选择从事护士工作，我感到高兴					
