# Supplementary material for: Analysis of the correlation between clinical nurses' professional quality of life and family care and organizational support
Source: Front Public Health. 2023 Feb 22;11:1108603. doi: 10.3389/fpubh.2023.1108603 (PMC9992405; doi:10.3389/fpubh.2023.1108603)
Supplement: Supplementary file 3 [file Table_3.pdf]

Supplementary Table 3. Organizational Support Scale.

Items	Completely disagree	Disagree	Neutral	Agree	Completely agree
1. The organization respects my goals and values					
2. The organization recognizes my contributions in my work					
3.The organization values my opinions or views					
4.The organization makes my work reflect my personal abilities and values as much as possible					
5.The organization cares about my welfare and benefits					
6.The organization cares about my physical and mental health					
7.The organization takes my interests into consideration when making decisions					
8.The organization will try to help me when I need special help (such as pregnancy, special family events or changes, etc.)					
9.The organization cares about my personal career development					
10. The organization will provide me with opportunities for promotion					
11. The organization tries its best to provide me with training and exchange learning opportunities					
12. The organization tries its best to provide me with a safe working environment					
13. The organization tries its best to provide me with the resources and information I need to work					
14. The organization tries its best to continuously improve working conditions to make my work more effective					
15.When I have problems at work (such as job changes, nursing errors, etc.), the organization will do its best to help me analyze and find countermeasures					

条目	完全不同意	不同意	中立	同意	完全同意
1 组织尊重我的目标和价值观					
2 组织认可我在工作中的贡献					
3 组织重视我的意见或看法					
4 组织尽可能让我的工作能体现个人能力和价值					
5 组织关心我的福利待遇					
6 组织关心我的身心健康					
7 组织在作决策时会考虑到我的利益					
8 当我需要特殊帮助时，组织会尽力帮助我（如怀孕、家庭特殊事件或变故等）					
9 组织关心我的个人职业发展					
10 组织会提供我晋升的机会					
11 组织尽力提供给我培训和交流学习机会					
12 组织尽力为我提供安全的工作环境					
13 组织尽力为我提供工作所需的资源信息					
14 组织尽可能不断改善工作条件，使我的工作更有效率					
15 当我在工作中出现问题时（如岗位变动、护理差错等），组织会尽力协助我分析、找出对策					
