# Supplementary material for: Analysis of the correlation between clinical nurses' professional quality of life and family care and organizational support
Source: Front Public Health. 2023 Feb 22;11:1108603. doi: 10.3389/fpubh.2023.1108603 (PMC9992405; doi:10.3389/fpubh.2023.1108603)
Supplement: Supplementary file 4 [file Table_4.pdf]

Supplementary Table 4. Work-Family Conflict Scale

	Completely disagree	Disagree	Neutral	Agree	Completely agree
1. The demands of my job interfere with my family life					
2. My work schedule makes it difficult for me to meet my share of family responsibilities					
3. I don't get to do what I want to do because of the work that is weighing on me					
4. The pressure of my job makes it difficult to change my family's schedule of activities					
5. I have to make changes to my family life plan because of my work responsibilities					
6. My family (or partner) makes demands that interfere with my work activities					
7. I had to postpone work due to family reasons					
8. I was not able to finish my work because my family (or partner) made some requests that took up my original work time					
9. My family life affects my work responsibilities, such as getting to work on time, completing daily tasks, and working overtime					
10. Family pressure affects my ability to perform in my work tasks					

Supplementary Table 4. Work-Family Conflict Scale

	非常不同意	不同意	不一定	同意	非常同意
1.我的工作要求影响了我的家庭生活					
2.我的工作时间使得我很难满足我应承担的家庭责任					
3.因为压在我身上的工作，我没能干完自己想做的事					
4.我的工作压力使得我很难更改我的家庭活动计划					
5.因为工作责任所在，我必须对我的家庭生活计划作出改动					
6.我的家人（或伴侣）提出的要求影响了我的工作活动					
7.由于家里的原因我不得不推迟工作					
8.我没能干完工作中的事情，是因为我的家人（或伴侣）提出了一些要求而占据了我原本的工作时间					
9.我的家庭生活影响了我的工作责任，比如按时上班、完成日常任务和超时工作					
10.家庭压力影响我在工作任务中能力的发挥					
