# Supplementary material for: Analysis of the correlation between clinical nurses' professional quality of life and family care and organizational support
Source: Front Public Health. 2023 Feb 22;11:1108603. doi: 10.3389/fpubh.2023.1108603 (PMC9992405; doi:10.3389/fpubh.2023.1108603)
Supplement: Supplementary file 5 [file Table_5.DOCX]

Supplementary Table 5. Reliability test of the scale used in this study

| Scale | Dimensionality | Cronbach's alpha |
| --- | --- | --- |
| Nurse Professional Quality of Life Scale | **—** | 0.931 |
|  | Compassion satisfaction | 0.821 |
|  | Burnout | 0.716 |
|  | Secondary trauma | 0.768 |
| Family Care Scale | **—** | 0.905 |
| Organizational Support Scale | **—** | 0.985 |
| Work-Family Conflict Scale | **—** | 0.903 |
