# Supplementary material for: Analysis of the correlation between clinical nurses' professional quality of life and family care and organizational support
Source: Front Public Health. 2023 Feb 22;11:1108603. doi: 10.3389/fpubh.2023.1108603 (PMC9992405; doi:10.3389/fpubh.2023.1108603)
Supplement: Supplementary file 6 [file Table_6.DOCX]

Supplementary Table 6. Scores on each scale in selected sections

|  | Internal Medicine | Surgery | Obstetrics & Gynecology | Pediatrics | Emergency | ICU | Oncology |
| --- | --- | --- | --- | --- | --- | --- | --- |
| FC | (7.12$\pm$2.63) | (7.28$\pm$2.64) | (7.36$\pm$2.80) | (7.50$\pm$2.55) | (6.91$\pm$2.55) | (6.80$\pm$2.23) | (7.04$\pm$2.75) |
| SC | (51.34$\pm$10.70) | (50.01$\pm$9.95) | (49.39$\pm$10.68) | (49.71$\pm$8.86) | (49.28$\pm$10.63) | (50.40$\pm$8.57) | (50.38$\pm$9.24) |
| Burnout | (49.67$\pm$11.01) | (50.79$\pm$9.90) | (50.59$\pm$10.24) | (48.98$\pm$9.39) | (48.71$\pm$9.27) | (49.19$\pm$8.95) | (50.75$\pm$9.03) |
| ST | (49.97$\pm$9.93) | (50.70$\pm$10.05) | (49.60$\pm$9.35) | (49.36$\pm$9.58) | (50.57$\pm$9.49) | (50.22$\pm$9.30) | (50.39$\pm$9.78) |
| LS | (21.69$\pm$5.68) | (22.10$\pm$6.21) | (22.23$\pm$6.10) | (21.98$\pm$6.40) | (22.03$\pm$5.61) | (21.39$\pm$5.48) | (22.18$\pm$6.16) |
| OS | (58.24$\pm$11.34) | (58.53$\pm$11.98) | (58.72$\pm$10.75) | (58.36$\pm$10.33) | (57.00$\pm$9.83) | (57.60$\pm$10.10) | (59.21$\pm$12.40) |
| SOC | (6.21$\pm$0.92) | (6.32$\pm$0.99) | (6.43$\pm$0.99) | (6.37$\pm$0.95) | (6.24$\pm$0.88) | (6.07$\pm$0.60) | (6.19$\pm$0.97) |
| WFC | (28.42$\pm$7.11) | (26.99$\pm$7.58) | (26.81$\pm$6.58) | (27.44$\pm$7.17) | (28.18$\pm$6.10) | (27.36$\pm$6.77) | (27.38$\pm$7.37) |

|  | General Section | Orthopedics | Infectious Diseases | Medical and Technical Departments | Operating room | Outpatient | Other | |
| --- | --- | --- | --- | --- | --- | --- | --- | --- |
| FC | (7.12$\pm$2.63) | (7.28$\pm$2.64) | (7.36$\pm$2.80) | (7.50$\pm$2.55) | (6.91$\pm$2.55) | (6.80$\pm$2.23) | (7.04$\pm$2.75) | |
| SC | (51.34$\pm$10.70) | (50.01$\pm$9.95) | (49.39$\pm$10.68) | (49.71$\pm$8.86) | (49.28$\pm$10.63) | (50.40$\pm$8.57) | (50.38$\pm$9.24) |  |
| Burnout | (49.67$\pm$11.01) | (50.79$\pm$9.90) | (50.59$\pm$10.24) | (48.98$\pm$9.39) | (48.71$\pm$9.27) | (49.19$\pm$8.95) | (50.75$\pm$9.03) | |
| ST | (49.97$\pm$9.93) | (50.70$\pm$10.05) | (49.60$\pm$9.35) | (49.36$\pm$9.58) | (50.57$\pm$9.49) | (50.22$\pm$9.30) | (50.39$\pm$9.78) | |
| LS | (21.69$\pm$5.68) | (22.10$\pm$6.21) | (22.23$\pm$6.10) | (21.98$\pm$6.40) | (22.03$\pm$5.61) | (21.39$\pm$5.48) | (22.18$\pm$6.16) | |
| OS | (58.24$\pm$11.34) | (58.53$\pm$11.98) | (58.72$\pm$10.75) | (58.36$\pm$10.33) | (57.00$\pm$9.83) | (57.60$\pm$10.10) | (59.21$\pm$12.40) | |
| SOC | (6.21$\pm$0.92) | (6.32$\pm$0.99) | (6.43$\pm$0.99) | (6.37$\pm$0.95) | (6.24$\pm$0.88) | (6.07$\pm$0.60) | (6.19$\pm$0.97) | |
| WFC | (28.42$\pm$7.11) | (26.99$\pm$7.58) | (26.81$\pm$6.58) | (27.44$\pm$7.17) | (28.18$\pm$6.10) | (27.36$\pm$6.77) | (27.38$\pm$7.37) | |

Abbreviation: SC: Compassion satisfaction; ST: Secondary trauma;FC: Family Care; LS: Life satisfaction; OS: Organizational Support; WFC: Work-family conflict; SOC: Sense of mental consistency
